# Efficacy of INtensive Treatment vs. Standard Treatment of COmpound DanshEn Dripping Pills in Refractory Angina Patients With Incomplete Revascularization (INCODER Study): Study Protocol for a Multicenter, Double-Blind, Randomized Controlled, Superiority Trial

**DOI:** 10.3389/fcvm.2022.860059

**Published:** 2022-04-26

**Authors:** Zexuan Wu, Danping Xu, Zhen Wu, Ailan Chen, Lijuan Liu, Li Ling, Yan Zhou, Duoduo Liu, Yin Liu, Yugang Dong, Yili Chen

**Affiliations:** ^1^Department of Cardiology, the First Affiliated Hospital of Sun Yat-sen University, Guangzhou, China; ^2^National Health Commision (NHC) Key Laboratory of Assisted Circulation (Sun Yat-sen University), Guangzhou, China; ^3^Department of Chinese Traditional Medicine, The Eighth Affiliated Hospital, Sun Yat-sen University, Shenzhen, China; ^4^Department of Cardiology, The Third Affiliated Hospital of Sun Yat-sen University, Guangzhou, China; ^5^Department of Cardiology, The First Affiliated Hospital of Guangzhou Medical University, Guangzhou, China; ^6^Department of Cardiology, The East Division of the First Affiliated Hospital, Sun Yat-sen University, Guangzhou, China; ^7^Department of Medical Statistics, School of Public Health, Sun Yat-sen University, Guangzhou, China; ^8^Department of Cardiology, Hainan General Hospital, Haikou, China; ^9^Department of Cancer Epidemiology, Henan Cancer Hospital, Affiliated Cancer Hospital of Zhengzhou University, Zhengzhou, China

**Keywords:** compound Danshen dripping pills, intensive treatment, efficacy, refractory angina, incomplete revascularization

## Abstract

**Introduction:**

Patients with incomplete revascularization (ICR) tend to develop refractory angina despite optimal medical therapy. The Compound Danshen Dripping Pills (CDDP) is a widely used antianginal drug in China and is shown to significantly alleviate myocardial ischemia. Previous studies showed dose-efficacy tendency when increasing doses of CDDP. This study aims to investigate the efficacy and safety of intensive doses of CDDP in patients with refractory angina with ICR.

**Methods and Analysis:**

The INCODER study is a multicenter, double-blind, randomized controlled, superiority trial. We plan to recruit 250 patients aged 18–85 years with a diagnosis of refractory angina with ICR. Patients will be randomized (1:1) to intensive treatment group (CDDP 20 pills three times per day) or standard treatment group (10 pills CDDP and 10 pills placebo three times per day). Patients will have a 6-week medication period and be followed up every 2 weeks. The primary endpoint is the change of total exercise time from baseline to week 6 as assessed by cardiopulmonary exercise testing (CPET). Secondary endpoints include changes in the frequency of angina, Canadian Cardiovascular Society angina class, nitroglycerin use, Seattle Angina Questionnaire scores, peak oxygen uptake (VO_2_ peak) and other parameters as measured by CPET, and the levels of plasma C-reactive protein, homocysteine, and N-terminal pro-B-type natriuretic peptide. Safety events related to CDDP use will be monitored.

**Ethics and Dissemination:**

The research had been approved by the Clinical research and laboratory animal ethics committee of the First Affiliated Hospital, Sun Yat-sen University ([2019]65). The results will be reported through peer-reviewed journals, seminars, and conference presentations.

**Trial Registration Number:**

www.chictr.org.cn (ChiCTR2000032384). Registered on 27 April 2020.

## Background

Multivessel coronary artery disease (CAD), which is defined as stenosis of >50% affecting more than one epicardial vessel, is found in about 50% of patients on diagnostic angiography and is significantly associated with a worse prognosis than single-vessel CAD (1, 2). Due to various reasons including old age, multiple comorbidities (particularly diabetes mellitus), and complex coronary lesions such as chronic total occlusions, bifurcation disease, diffuse disease or narrow segments, and multiple lesions, about 43.3% of the patients who underwent percutaneous coronary intervention (PCI) and 36.8% of patients who underwent coronary artery bypass grafting (CABG) could not achieve complete revascularization (CR) (2, 3). According to the New York's PCI registry that included 41,639 New York residents with multivessel coronary artery disease undergoing PCI, the rate of incomplete revascularization (ICR) was up to 78% among patients with ST-segment elevation myocardial infarction and 71% among other patients (4). These patients were associated with recurrent cardiovascular events, frequent hospital re-admissions, and decreased quality of life (5, 6). Despite optimal medical therapy (OMT), up to 10–15% of patients with CAD develop refractory angina with an annual mortality rate as high as 4% (7–9). CR may be a choice for patients who are in good condition; however, recent studies have shown controversial results on the prognosis benefit of PCI over OMT in stable patients (10, 11). Therefore, there is an unmet need for additional medical strategies to alleviate anginal symptoms and improve the prognosis for patients with ICR. The European Society of Cardiology (ESC) guidelines recommend using ranolazine, ivabradine, nicorandil, or trimetazidine as a second-line therapy for patients with persistent angina according to heart rate, blood pressure, and tolerance (class IIa, level of evidence B) (9). However, the placebo-controlled randomized Ranolazine in patients with Incomplete Revascularization after Percutaneous Coronary Intervention (RIVER-PCI) trial showed no incremental benefits in improving angina symptoms, quality of life, or prognosis for patients with ICR by adding ranolazine (12, 13). Evidence was also lacking on the efficacy of ivabradine, trimetazidine, or nicorandil in patients with ICR (14). Consequently, robust randomized and controlled trials that aim to achieve more effective pharmacological agents for these challenging populations are urgently needed.

Compound Danshen dripping pills (CDDP) or Dantonic, an herbal patent medicine, is widely used in China for the clinical treatment of CAD. CDDP contains three medicinal herbs, *Salvia miltiorrhiza*, notoginseng, and borneol at a ratio of 450:141:8 (15). Its main active pharmacodynamic substances are phenolic acids, saponins, and borneol (16). According to previous studies, CDDP played an important role in the management of CAD through regulating multiple targets that were associated with thrombosis, hyperlipidemia, and vascular remodeling (17). A meta-analysis comprising of 60 randomized controlled clinical trials, including a total of 6,931 patients, showed that CDDP is apparently more effective than isosorbide dinitrate in the treatment of angina pectoris (18). Another systematic review analysis including 34,071 patients with CAD (including patients with angina pectoris and acute myocardial infarction) confirmed the benefits of CDDP in these patients (19). The recommended dose for CAD is 10 pills, three times per day in China (16). In the Phase II clinical trial of CDDP conducted under the supervision of Food and Drug Administration (FDA) in the United States, 125 patients with moderate chronic stable angina pectoris were randomly divided into three groups: placebo, low-, or high-dose group (0, 20, or 30 pills, twice per day, respectively). The results indicated a significant dose-response relationship regarding total exercise duration (TED) following the standard Bruce protocol. After 4 weeks of treatment, the mean improvement of TED compared to placebo was 20 s in the low-dose group (*p* = 0.18) and 43 s in high-dose group (*p* = 0.005). No serious adverse drug reactions (ADRs) were observed (16). Although the changes of TED between low- and high-dose groups were not significantly different, a tendency for better improvement of TED was observed in the higher dose treatment group. For patients with refractory angina with ICR, we found a dose-efficacy tendency when the dose was increased to 20 pills three times per day in clinical practice. Considering more serious conditions, no options or refusal for revascularization, poor response to traditional medical treatment, and different races and nationalities compared with FDA phase II clinical trial, we designed this study and inferred that intensive doses of CDDP would be better for the improvement of exercise capacity in patients with refractory angina without CR.

As for the safety issue, according to the reports from China FDA (CFDA) center for ADRs, the incidence rate of ADRs for CDDP is only 0.0018%, most of which were gastrointestinal symptoms, non-specific hemorrhage, and non-specific purpura (16). Most of the ADRs were mild and reversible.

Therefore, we designed the study to evaluate the efficacy and safety of INtensive treatment *vs*. standard treatment of COmpound DanshEn dripping pills in Refractory angina patients with ICR (INCODER study).

## Methods and Analysis

### Study Design

**Figure 1:**
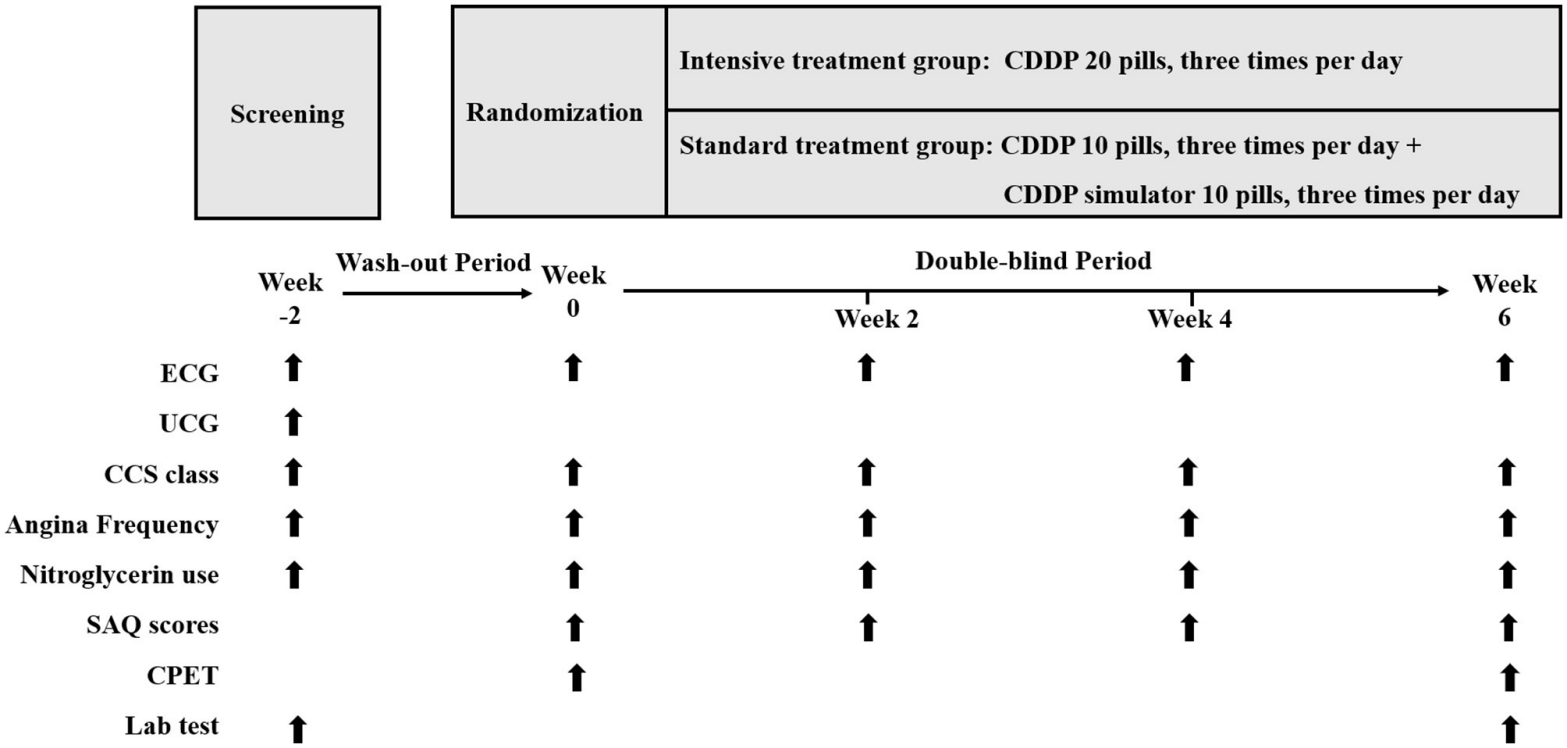
Study design. CDDP, Compound Danshen Dripping Pills; ECG, electrocardiogram; UCG: echocardiography; CCS, Canadian Cardiovascular Society; SAQ, Seattle Angina Questionnaire; CPET, cardiopulmonary exercise testing.

The INCODER study is a multicenter, randomized, double-blind, parallel controlled, superiority clinical trial. Patients will be randomly divided into two groups: intensive treatment group (CDDP 20 pills three times per day) or standard treatment group (CDDP 10 pills three times per day). Figure 1 shows the design and procedures through the trial.

### Study Hypothesis

The administration of CDDP 20 pills three times per day is superior to 10 pills three times per day with respect to efficacy and safety in patients with refractory angina with ICR.

### Study Population, Eligibility Criteria, and Recruitment

Patients aged 18–85 years with a diagnosis of stable refractory angina with ICR are eligible for participation. Patients are eligible for inclusion in the study if they had a history of CAD with refractory angina (Canadian Cardiovascular Society [CCS] class II to III) despite OMT for at least 1 month. OMT is defined as a guideline-recommended treatment including secondary prevention and at least two antianginal drugs: one is nitrates and the other is a beta-blocker or calcium channel blocker, except for medication contraindications (20). ICR is defined as the presence of ≥1 lesion with visually estimated ≥50% diameter stenosis in any coronary artery (including branch vessels) of ≥2.0 mm in diameter, whether in the target vessel or a non-target vessel. In the case of a participant post-CABG, ICR is defined as the presence of ≥1 lesion with visually estimated ≥50% diameter stenosis in a non-bypassed epicardial vessel ≥2.0 mm in diameter, or ≥1 visually estimated ≥50% diameter stenosis in a bypass graft supplying an otherwise non-revascularized myocardial territory (21). The ejection fraction should be at least 30%. Patients should be willing to sign the informed consent form.

The exclusion criteria are detailed in Table 1. Patients with acute coronary syndrome within 1 month or planned coronary revascularization during the study period or a history of transient ischemia attack within 2 months are excluded. Patients with severe joint diseases, severe peripheral vascular disease, severe pulmonary hypertension, uncontrolled chronic obstructive pulmonary disease, and other comorbidities that might affect the results of the cardiopulmonary exercise testing (CPET) are excluded. We also exclude patients with abnormalities in electrocardiogram (ECG) at rest including preexcitation syndrome ventricular pacing rhythm, ST-segment depressed more than 1 mm in the rest, left bundle branch block or any intraventricular conduction block, and QRS duration more than 120 ms that might influence the interpretation of the termination of CPET. Patients with severe comorbidity, pregnant, or lactating are also excluded. Study candidates will be assessed for eligibility within 2 weeks prior to enrolment.

**Table 1:** Eligibility criteria.

**Inclusion criteria** 1. Age 18 to 85 years. 2. Had a history of coronary artery disease with refractory angina [Canadian Cardiovascular Society (CCS) class II to III] despite optimal medical therapy for at least 1 month. - Optimal medical therapy is defined as guideline-recommended treatment including secondary prevention and at least two antianginal drugs: one is nitrates, another is beta-blocker or calcium channel blocker, except for medication contraindications. 3. With incomplete revascularization (ICR). - ICR is defined as the presence of ≥1 lesion with visually estimated ≥50% diameter stenosis in any coronary artery (including branch vessels) of ≥ 2.0 mm in diameter, whether in the target vessel or in a non-target vessel. In the case of a participant post-CABG, ICR is defined as the presence of ≥1 lesion with visually estimated ≥50% diameter stenosis in a non-bypassed epicardial vessel ≥2.0 mm in diameter, or ≥ 1 visually estimated ≥ 50% diameter stenosis in a bypass graft supplying an otherwise non-revascularized myocardial territory. 4. The ejection fraction is ≥ 30%.^a^ 5. Willing to sign the informed consent form. **Exclusion criteria** 1. Acute coronary syndrome within 1 month or planned coronary revascularization during the study period; 2. Episode of transient ischemia attack, ischemic or hemorrhagic stroke within 2 months; 3. Presence of neuromuscular, orthopedic or other non-cardiac condition such as severe peripheral artery disease, severe pulmonary hypertension and uncontrolled chronic obstructive pulmonary disease or asthma that prevents the patient from exercise testing on a cycle ergometer; 4. Patients with the following resting electrocardiographic abnormalities that might influence the interpretation of the termination of CPET: - Pre-excitation (Wolff-Parkinson-White) syndrome - Electronically paced ventricular rhythm - 1 mm or more of resting ST depression - Complete left bundle-branch block or any interventricular conduction defect with a QRS duration >120 ms 5. Uncontrolled symptomatic heart failure; 6. Moderate to severe symptomatic aortic stenosis; 7. Hypertrophic cardiomyopathy; 8. Acute myocarditis or pericarditis; 9. Active endocarditis; 10. Acute pulmonary embolism; 11. Suspected or known acute aortic dissection; 12. Severe hypertension (systolic blood pressure > 180 mmHg or diastolic blood pressure > 110 mmHg at screening); 13. Severe anemia (Hemoglobin <60 g/L); 14. Active psychosis requiring anticonvulsant treatment; 15. Pregnant or lactating women. 16. Participation in other clinical studies within 30 days before the first visit.

*CABG, coronary artery bypass grafting; CPET, cardiopulmonary exercise testing*.

a *Echocardiographic results measured with 3 months before obtaining informed consent can be used for assessing eligibility*.

The study will be conducted in 7 medical centers in China. Patients will be recruited by local physicians using posters and advertisements in the hospital and nearby communities. Posters and advertisements contain brief introductions about the study and the contact information of the investigators. The study will be explained in detail to the potential participants, and informed written consent will be obtained prior to enrolment.

### Randomization, Allocation, and Blinding

Randomization is generated using computer-generated random permuted blocks by a designated statistician not involved in study operations using SAS V9.4 software. Either CDDP or placebo will be packaged identically and labeled with a number according to the randomization list. The treatment kit numbers will be obtained by the investigators at the time of patients' randomization, and treatment kits will be strictly sequentially allocated to patients according to the randomization list. In accordance with the double-blind design, the sponsor, study patients, investigators, and study site personnel will remain blinded to study treatment.

The randomization code will be kept in opaque sealed envelopes and secured by both the sponsor and the principal investigator and can only be broken in exceptional circumstances when knowledge of the investigational drug is essential for treating the patient. If the randomization code is uncovered, the investigator should document the reason for unblinding, and the administration of the investigational drug will be discontinued.

### Intervention

The intensive treatment group is administered with CDDP (produced by Tasly Pharmaceutical Group Co. Ltd., China) at a dose of 20 pills three times per day while the standard treatment group takes a standard dose of 10 pills CDDP and 10 pills placebo three times per day orally after meals. All the patients will be treated for 6 weeks. CDDP or placebo pills are identical in size and smell. All the 20 pills (20 pills CDDP for intensive treatment group or 10 pills CDDP and 10 pills placebo for the standard treatment group) are stored in one identical bottle. Patients take one bottle three times per day.

### Concomitant Medication

Study participants should be treated with standard CAD therapies as per recommended guidelines. The following drugs are prohibited during the whole study including ivabradine, ranolazine, nicorandil, trimetazidine, and other antianginal Chinese traditional medicine (including ginkgo biloba dropping pill, ginkgolide dropping pill, Shexiang Baoxin Pill, Xinkeshu tablet, Naoxintong, Naoxinqing, xueshuantong, Di'ao xinxuekang capsule, Yinzhan xinmai dropping pill, Xinnaoxin, Zhenyuan capsule, Suxiao Jiuxin Pill, Tongxinluo, Guanxin Danshen Dropping Pill, yindanxintai dropping pill, etc.). However, other kinds of medications are taken as needed. The concomitant medication will be recorded throughout the trial and analyzed if necessary.

### Outcomes

#### Primary Outcome

The primary outcome is the change in total exercise time evaluated by CPET from baseline to week 6 in the intention-to-treat (ITT) population.

#### Secondary Outcomes

The secondary outcomes include changes from baseline to week 6 in the following parameters including the peak oxygen uptake (VO_2_ peak), frequency of angina, nitroglycerin use, CCS angina class improvement, Seattle Angina Questionnaire (SAQ) scores, other CPET indexes, as well as plasma C-reactive protein (CRP), homocysteine, and N-terminal pro-B-type natriuretic peptide (NT-proBNP) levels. Safety assessment includes adverse events during the whole study period, vital signs, physical examination, 12-lead ECG, laboratory tests, and the use of rescue medication (Table 2).

**Table 2:** Efficacy and safety endpoints.

**Primary endpoint** The changes in total exercise time evaluated by CPET from baseline to week 6 **Secondary endpoint** The changes of the following parameters from baseline to week 6 1. Peak VO_2_ (ml/min) 2. Peak VO_2_ (ml/min/kg) 3. Frequency of angina 4. Frequency of nitroglycerine use 5. Proportion of patients experiencing an improvement of ≥1 CCS angina classes 6. Proportion of patients experiencing an improvement of ≥2 CCS angina classes 7. The SAQ scores subdivided in the following 5 dimensions - Physical limitation; - Anginal stability; - Anginal frequency; - Treatment satisfaction; - Disease perception. 8. Other CPET parameters - VO_2_ at anaerobic threshold (ml/min) - VO_2_ at anaerobic threshold (ml/min/kg) - Peak METs - Peak work rate (W) - O_2_ pulse at peak exercise (mL/beat) - P_ET_O_2_ at peak exercise (mmHg) - P_ET_CO_2_ at peak exercise (mmHg) - VE/VCO_2_ slope - ΔVO_2_/Δwork-rate (ml/min/W) 9. Plasma CRP, homocysteine and NT-proBNP **Safety endpoints** 1. Treatment-emergent adverse event 2. Vital signs 3. Physical examination 4. ECG 5. Clinical laboratory tests

*CPET, cardiopulmonary exercise testing; CCS, Canadian Cardiovascular Society; SAQ, Seattle Angina Questionnaire; VO_2_, Oxygen uptake; METs, metabolic equivalent levels; P_ET_O2, partial pressure of end-tidal oxygen; P_ET_CO2, partial pressure of end-tidal carbon dioxide; VE/VCO_2_, Ventilatory equivalent of carbon dioxide; CRP, C-reactive protein; NT-proBNP, amino-terminal pro-B-type natriuretic peptide; ECG: electrocardiography*.

### Study Timelines

#### Screening Visit (Week-2)

The schedule of enrolment and assessments are listed in Table 3. Subjects will be evaluated at the screening visit 2 weeks prior to enrolment through a complete medical history, vital signs, physical examination, 12-lead ECG, transthoracic echocardiography (TTE), eligibility laboratory tests, and a review of concomitant medications. Eligibility and baseline laboratory examinations include urine pregnancy testing for women with childbearing potential, complete blood count, serum lipids, biochemical tests, coagulation test and cardiac biomarkers. Baseline parameters for efficacy evaluation including CRP, homocysteine, and NT-proBNP will also be examined. TTE measured 3 months before obtaining informed consent can be used for eligibility assessment. The severity of angina will be assessed using CCS angina class, frequency of angina attack, and nitroglycerin use.

**Table 3:** Schedule of assessments.

**Visit number**	**V1**	**V2**	**V3**	**V4**	**V5**
Week	−2	0	2	4	6
Day	−14 ± 3	0	14 ± 3	28 ± 3	42 ± 3
Informed consent	×				
Inclusion/ exclusion criteria	×	×			
Medical history	×	×			
Concomitant medication	×	×	×	×	×
Vital signs	×	×	×	×	×
Physical examination	×	×	×	×	×
urine pregnancy testing^a^	×				
12-lead ECG	×	×	×	×	×
Transthoracic echocardiography^b^	×				
CCS class	×	×	×	×	×
SAQ scores		×	×	×	×
Frequency of angina	×	×	×	×	×
CPET		×			×
Laboratory tests^c^	×				×
Dispense study medication and collect empty study medication		×	×	×	×
Dispense nitroglycerin and collect the remaining pills;	×	×	×	×	×
Compliance evaluation		×	×	×	×
Adverse events	×	×	×	×	×

*ECG, electrocardiogram; CCS, Canadian Cardiovascular Society; SAQ, Seattle angina questionnaire; CPET, cardiopulmonary exercise testing*.

a *Urine pregnancy testing for women with childbearing potential for eligibility*.

b *Echocardiographic results measured with 3 months before obtaining informed consent can be used for assessing eligibility*.

c *Laboratory tests for efficacy and safety include:*

*-Complete blood count*

*-Serum lipids*

*-Biochemical indexes: alanine aminotransferase (ALT), aspartate aminotransferase (AST), lactate dehydrogenase (LDH), creatine kinase (CK) and creatinine*

*-Coagulation indexes: prothrombin time (PT), partially activated prothrombin time (APTT), international normalized ratio (INR), coagulation time (TT), fibrinogen (FBG) and D-dimer*

*-Cardiac biomarkers: myoglobin (MYO), cardiac troponin T (cTnT) and creatine kinase-MB isoenzyme (CK-MB)*

*-Parameters for efficacy: C-reactive protein (CRP), homocysteine and amino-terminal pro-B-type natriuretic peptide (NT-proBNP)*.

#### Wash-Out Period

Patients who are taking other antianginal drugs that are contraindicated in our study at screening will be asked to start a 2-week wash-out period, in which they will discontinue the antianginal drugs and follow the OMT. Nitroglycerin is allowed during the wash-out period and should be recorded when used.

#### Baseline Visit (Week 0)

Research participants will complete all baseline procedures, including clinical evaluation, angina assessment, SAQ scores, and CPET. Patients who fulfill all the inclusion criteria and none of the exclusion criteria will be randomized with a 1:1 allocation ratio to receive either CDDP 20 pills or 10 pills three times per day. Nitroglycerin will also be dispensed for angina attack when needed. Patients are asked to record every angina attack and nitroglycerin consumption during the study.

#### Follow-Up Visit (Week 2 and Week 4)

Patients will be followed up every 2 weeks to assess safety and tolerability. Vital signs, physical examination, angina assessment, and SAQ scores will be recorded. Any adverse events during the intervening period from screening/baseline visit will be recorded. Study drug dispensed at screening/baseline visit will be collected for compliance calculation.

#### Final Evaluation (Week 6)

Final evaluations will be conducted 6 weeks following the baseline assessment. Vital signs, physical examination, 12-lead ECG, angina assessment, SAQ scores, laboratory analyses, and adverse events will be assessed. Blood analyses including CRP, homocysteine, and NT-proBNP for efficacy assessments and other parameters for safety assessments consistent with baseline will be performed. CPET will be conducted for efficacy evaluation.

### Data Collection, Management, and Analysis

#### Sample Collection and Laboratory Measurements

Blood sample collection is scheduled at screening/randomization and the final visit. Blood samples are first collected into evacuated tubes, allowed clot for 30 min at room temperature, and then centrifugated for 10 min at 3,000 × *g* at room temperature. Beckman Coulter AU5800 chemistry analyzer is used for further measurements. Urine samples will also be collected at screening for a urine pregnancy test for women with childbearing potential. All the laboratory measurements mentioned above will be performed at the Department of Laboratory Medicine of each site with consistent external quality control.

#### Angina Assessment

The CCS angina class, SAQ scores, frequency of angina attack, and nitroglycerin use will be recorded for angina assessment during the whole study period. The SAQ scores measure five important dimensions of CAD including physical limitation, anginal stability, anginal frequency, treatment satisfaction, and disease perception (22).

#### Cardiopulmonary Exercise Testing

The CPET is conducted on an electronically braked cycle ergometer (Ergoline GmbH, Germany) using a ramp protocol according to the statement from the American Heart Association (23, 24). Expired gases are collected and analyzed breath by breath. The test includes several stages (23): (1) rest for 3 min: measurements of rest ECG, blood pressure, heart rate and pulse oxygen saturation; (2) unloading exercise for 3 min; (3) incremental exercise. The increment rate is set between 10 and 20 W·min^−1^ based on patients' characteristics to obtain ~10 min duration. The resistance gradually increases every 6 s. All patients are instructed to cycle with a pedal frequency of 60 revolutions per minute (rpm) and are encouraged to keep exercising until exhaustion or symptoms of chest tightness, chest pain, and shortness of breath, or inability to maintain the pedal frequency of 60 rpm. (4) Recovery: unloading exercise for 3 min, then rest for 5–10 min. The blood pressure is measured by using Tango M2 Stress Test Monitor. The heart rate is determined from the electrocardiogram and expressed as the percentage of the predicted value (220—age). The 12-lead electrocardiogram is continuously recorded during the test. Symptoms are assessed by means of the Borg's Rating of Perceived Exertion (RPE, Borg's 6–20 Scale) (25). Peak exercise is considered the highest VO_2_ achieved during active exercise or early recovery. Heart rate and respiratory exchange ratio (RER) are objective indicators for peak exercise. Peak VO_2_ was determined as the highest mean VO_2_ over 20 s (24). Parameters measured during CPET in our study are listed in Supplementary Table 1. The indications of termination of the test are detailed in Supplementary Table 2. The total exercise time and reason for termination are recorded.

#### Compliance Evaluation

Compliance will be evaluated by counting the number of bottles returned at every follow-up visit (total doses taken divided by total prescribed doses). Of note, the denominator (total prescribed doses) will be calculated over the actual duration when the subject is in the study. Subjects will be informed of the intention to monitor compliance in the informed consent form.

#### Adverse Events and Safety

Adverse events will be assessed and recorded in the case report forms during the whole study. Given the nature of the intervention, the most common adverse reactions related to CDDP including gastrointestinal symptoms, headache, and facial flushing will be monitored. Although CPET is fairly safe with a serious complications rate of <1–5 per 10, 000 tests and a death rate of around 0.5 per 10,000 tests, complications do occur and should be paid attention (24, 26). Major complications include death, myocardial infarction, angina pectoris, arrhythmia, hemodynamic instability, and orthopedic injury (21). Therefore, the test should strictly follow the guideline for exercise stress testing especially the indications to stop the test (Supplementary Table 2).

All serious adverse events (SAEs) will be assessed and reported to the ethics committee in accordance with the International Council for Harmonization of Technical Requirements for Registration of Pharmaceuticals for Human Use Good Clinical Practice (GCP) guidelines (27).

#### Data Management and Monitoring

Data will be collected by trained research staff at each site and recorded in case report forms. This will then be entered into the INCODER database, which is located in the clinical research unit in the Department of Cardiology in the First Affiliated Hospital, Sun Yat-sen University, and supervised by the trial data manager. Data from the INCODER study will be transferred securely to the Research Data Deposit at http://www.researchdata.org.cn/, where it can be accessed and traced securely *via* a specialized administration panel by members of the research team using an encrypted password.

The INCODER study will be overseen by the trial steering committee (TSC) in accordance with the GCP guidance. The TSC, which consists of the chief investigator, study coordinator, site investigators, and statisticians, will independently monitor the process of the study and give advice on the continuation, termination, or amendments of the trial protocol. The study is sponsored by Tasly Pharmaceutical Group Co. Ltd. and will be subject to regular monitoring visits and audits.

#### Patient and Public Involvement

Patients were neither involved in the design or the recruitment and conduct of the study, nor in the assessment of the burden of the intervention. Results will be disseminated to participants on request.

### Statistical Consideration

#### Sample Size Assumptions

This is a superiority study. Sample size calculation is based on the primary outcome of change in total exercise time from baseline to follow-up at 6 weeks. According to the unpublished results of Phase II clinical trial conducted in the United States (16) and our preliminary study (http://www.chictr.org.cn; Registration number: ChiCTR-IIR-17013662), the change of total exercise time is 62.5±19.6 s and 54.5±18.8 s from baseline to week 6 in intensive treatment group and standard treatment group, respectively. We, therefore, assumed the changes of total exercise time as 62.5 s and 54.5 s in the two groups, respectively, and combined standard deviations as 20. The sample size was calculated based on the following formula


$$\begin{array}{l} {\text{N}}_{\text{c}}\;=\;\frac{{\left(Z_{1\;-\;\alpha }\;+\;Z_{1\;-\;\beta }\right)}^{2}\sigma^{2}\left(1\;+\;\frac{1}{r}\right)}{{\left(\mu_{E}\;-\;\mu_{C}-△\right)}^{2}}\\ {\text{N}}_{\text{t}}\;=\;r{\text{N}}_{c} \end{array}$$

where *N*_*c*_ and *N*_t_ denote the sample size of the standard treatment group and the intensive treatment group, respectively. In the equation, σ is the combined standard deviation, and α and β are type I and type II errors, respectively. Here, *r* is the allocation ratio between the two groups, and μ_*E*_ and μ_*C*_ are the average changes of the total exercise time before and after treatment among the intensive treatment group and the standard treatment group, respectively. The superiority margin is Δ. In our study, α = 0.025, β = 0.2, μ_*E*_ − μ_*C*_ = 62.5–54.5 = 8, σ ≈ 20, *r* = 1, and Δ = 0. A sample size of 100 per group will yield 80% power to obtain a significant difference between the 2 groups, using a 2-sample *t*-test at a 1-sided 0.025 level of significance. Considering an ~20% dropout rate, a total of 250 subjects (125 subjects for each group) will be enrolled.

### Statistical Analysis

Statistical analysis will be conducted by the SAS V9.4 software using a two-tailed 0.05 significance level.

Baseline demographic characteristics of the two groups will be presented using descriptive statistics. Continuous variables will be presented with median and interquartile range or mean ± SD. Categorical variables will be expressed as frequencies and percentages. For comparisons between the two groups, *t*-test or rank sum test will be used for continuous variables and the chi-square or Fisher's exact test will be applied for categorical variables.

The primary outcome, changes of total exercise time between the intensive treatment group and the standard treatment group, will be estimated based on the ITT population consisting of all the randomized patients. The general linear model will be used for the primary outcome with covariates of center and baseline exercise time using a 1-sided 0.025 significance level. Data will be presented as mean differences and corresponding *p-*values. Missing data will be imputed by using the last observation carried forward method.

We will further conduct ITT and per-protocol analysis to evaluate the differences in certain secondary outcomes between the two groups. Safety analyses will also be conducted based on a safety set in which all randomized subjects receive ≥1 dose of study medication.

### Ethics and Dissemination

The protocol was approved by the Clinical Research and Laboratory Animal Ethics Committee of the First Affiliated Hospital, Sun Yat-sen University ([2019]65), and all participants provided their written informed consent. All study procedures were followed with the ethical standards of the Helsinki Declaration. The study protocol was registered at www.chictr.org.cn (ChiCTR2000032384) prior to trial commencement.

Changes to the study protocol are documented in amendments. Amendments are submitted for approval to the Ethics Committee of the First Affiliated Hospital, Sun Yat-sen University. Major changes will be updated on the trial registration website of www.chictr.org.cn.

After completion, the results will be published in a peer-reviewed journal.

## Discussion

Over the past decades, there have been great advances in interventional therapy and medical therapy in CAD. However, still a large proportion of patients cannot achieve CR owing to patient comorbidities, anatomical factors, and technical or procedural considerations (28, 29). Despite OMT with anti-anginal drugs, patients complain of daily or weekly angina with the proportion ranging from 2 to 24% (30). Patients with long-lasting symptoms over 3 months that cannot be controlled by escalating medical therapy with the use of 2nd–and 3rd line pharmacological agents, PCI, or CABG are defined as refractory angina (31). In most studies, ICR after PCI or CABG was associated with poor prognosis (28, 32). Even for the patients with stable coronary disease and moderate or severe ischemia who have an opportunity for revascularization, recent studies found no evidence that an initial invasive strategy, as compared with an initial conservative medical therapy, reduced the risk of ischemic cardiovascular events or all-cause mortality (10, 11). Considering the growing “no-option” in patients who are inadequately responsive to “conventional” medical management and the limitations of invasive intervention, novel pharmacologic therapeutic strategies aiming at the improvement of angina symptoms, quality of life, and overall cardiac prognosis are of important clinical significance.

The CDDP is a traditional Chinese herb patent medicine that has been demonstrated to be effective in patients with CAD. In the FDA Phase II clinical trial conducted in the United States, there was a dose-response tendency in the mean improvement of total exercise duration in the high-dose group and low-dose group when compared with placebo (16). In clinical practice, we also found that intensive treatment of CDDP was better than the traditional dose with regard patients with refractory angina with ICR. However, robust randomized clinical trials are in great need. To be noted, the study population in our study are those without CR but still complaining of angina, which usually has more commodities and is much worse than those in the FDA Phase II clinical trial in the United States. Moreover, considering the differences in races and patients' conditions, we infer that an intensive dose of CDDP may bring extra benefits. To evaluate the feasibility of our study, we conducted a preliminary study aimed at the patients with refractory angina who were unsuitable for revascularization (http://www.chictr.org.cn; Registration number: ChiCTR-IIR-17013662). However, due to the difficulties in the patients' recruitment and enrollment, we expanded the scope of the inclusive patients to those with ICR. During the preliminary study, we also found it very important to unify the criteria for termination of the CPET test. Therefore, we optimized the termination criteria (Supplementary Table 2) and quality control of the test, making sure that the termination was symptom- or ischemia-limited, but not patient-subjective to stop.

Of note, the gold standard to evaluate exercise tolerance is treadmill exercise testing (33). However, for the treadmill exercise testing, patients are passively moving and may not be able to keep up with the speed of the treadmill or fear falling. As for the CPET, patients are encouraged to exercise to the limit and patients are allowed to stop by themselves when emergency. Therefore, our study adopts a relatively safe test of CPET to evaluate the exercise capacity of high-risk patients. Furthermore, CPET enables us to collect more fitness and metabolic parameters including peak VO_2_, METs, and work load, from which we can have a better understanding of patients' cardiopulmonary function and exercise tolerance. Our study may provide high-quality clinical evidence for the management of refractory angina patients without CR.

### Trial Status

Patient recruitment was started in August 2020 according to the protocol of version 4.0, 05-11-2019. Due to the COVID-19 pandemic, the recruitment and enrollment of the participant were slightly delayed than expected. Up to December 2021, a total of 54 patients had been enrolled. The study is expected to conclude in June 2023.

## Ethics Statement

The studies involving human participants were reviewed and approved by The Clinical research and laboratory animal Ethics Committee of the First Affiliated Hospital, Sun Yat-sen University ([2019]65). The patients/participants provided their written informed consent to participate in this study.

## Author Contributions

YD and YC conceived and instructed the study. ZW, DX, and ZW designed the study. LLin and YL provided statistical expertise and supported the development of the statistical analysis plan. AC, LLiu, YZ, and DL contributed to recruitment, trial oversight, intervention implementation, and the follow-up of the subjects. All the authors contributed to the design and development of the study protocol and have reviewed the manuscript.

## Funding

The INCODER study was sponsored by Tasly Pharmaceutical Group Co. Ltd (Grant number: K0601461). The study design, data collection, statistical analysis, or publications are not influenced by the sponsors and are exclusive responsibility of the investigators.

## Conflict of Interest

The authors declare that the research was conducted in the absence of any commercial or financial relationships that could be construed as a potential conflict of interest.

## Publisher's Note

All claims expressed in this article are solely those of the authors and do not necessarily represent those of their affiliated organizations, or those of the publisher, the editors and the reviewers. Any product that may be evaluated in this article, or claim that may be made by its manufacturer, is not guaranteed or endorsed by the publisher.
