# Supplementary material for: Efficacy of INtensive Treatment vs. Standard Treatment of COmpound DanshEn Dripping Pills in Refractory Angina Patients With Incomplete Revascularization (INCODER Study): Study Protocol for a Multicenter, Double-Blind, Randomized Controlled, Superiority Trial
Source: Front Cardiovasc Med. 2022 Apr 26;9:860059. doi: 10.3389/fcvm.2022.860059 (PMC9088738; doi:10.3389/fcvm.2022.860059)
Supplement: Supplementary file 1 [file Data_Sheet_1.docx]

**Supplementary Table 1. Parameters recorded during CPET**

| Variables |
| --- |
| Heart rate (per min) |
| Rest (seated) |
| Peak |
| Peak expressed as % of predicted |
| At ischemic or angina threshold |
| Blood pressure (mmHg) |
| Rest (seated) |
| Peak |
| Test increment rate (W·min^-1^) |
| Total exercise time (s) |
| Reason for test termination |
| Peak |
| Peak RER |
| Peak RPE |
| Peak VO_2_ (ml/min) |
| Peak VO_2_ (ml/min/kg) |
| Peak VO_2_ expressed as % of predicted |
| Peak METs |
| Peak Work rate (W) |
| O_2_ pulse at peak exercise (mL/beat) |
| P_ET_O_2_ at peak exercise (mmHg) |
| P_ET_CO_2_ at peak exercise (mmHg) |
| VO_2_ at anaerobic threshold (ml/min) |
| VO_2_ at anaerobic threshold (ml/min/kg) |
| V_E_/V_CO2_ slope |
| ΔVO_2_/Δwork-rate (ml/min/W) |

CPET, cardiopulmonary exercise testing; RER, respiratory exchange ratio; RPE, Rating of Perceived Exertion; VO_2_, Oxygen uptake; METs, metabolic equivalent levels; P_ET_O_2_, partial pressure of end-tidal oxygen; P_ET_CO_2_, partial pressure of end-tidal carbon dioxide; V_E_/V_CO2_, Ventilatory equivalent of carbon dioxide.

**Supplementary Table 2. Indications for Terminating CPET**

| 1. ST-segment elevation ≥1 mm persisting ≥1 minute 2. horizontal or downsloping ST-segment depression ≥1 mm persisting ≥1 minute 3. Symptom of angina, short of breath, wheezing 4. Arrhythmias including sustained ventricular tachycardia, multifocal PVCs, triplets of PVCs, supraventricular tachycardia, second or third atrioventricular block or new-onset bundle branch block 5. Increasing nervous system symptoms such as ataxia, dizziness or near-syncope 6. Signs of poor perfusion (cyanosis or pallor) 7. Drop in systolic blood pressure of >10 mmHg from baseline blood pressure despite an increase in work load 8. Drop in heart rate of ≥20 beats/min compared with the baseline accompanied by other signs of myocardial ischemia 9. Hypertensive response (systolic blood pressure >230 mmHg and/or diastolic blood pressure >115 mmHg) 10. Patients complaint of extremely fatigue or leg cramps with the pedal frequency of <40 r/min 11. Subject’s desire to stop |
| --- |

CPET, cardiopulmonary exercise testing; PVC, premature ventricular contraction.
